# Estimating abundance of harvested populations at the management unit scale

**DOI:** 10.1371/journal.pone.0326454

**Published:** 2025-06-18

**Authors:** Allison C. Keever, James D. Kelly, Garrett B. Clevinger, Bradley S. Cohen

**Affiliations:** 1 College of Arts and Sciences, Tennessee Technological University, Cookeville, Tennessee, United States of America; 2 Tennessee Wildlife Resources Agency, Ellington Agricultural Center, Nashville, Tennessee, United States of America; US Geological Survey, UNITED STATES OF AMERICA

## Abstract

Management of harvested populations relies on accurate assessment of abundance within management units to reevaluate and set harvest regulations. Several statistical approaches use readily available age-at-harvest data to estimate populations, but these often rely on auxiliary data which can be costly to collect and may not provide reliable estimates at the management unit scale. We developed a Bayesian integrated population model (IPM) relying solely on available harvest data to estimate abundance of white-tailed deer in Tennessee where estimates of abundance were lacking. We fit the IPM to reported harvest data and estimates of total harvest from hunter surveys to estimate abundance statewide and within deer management units (DMUs). Statewide deer harvest in Tennessee from 2005 to 2023 ranged between 132,256 and 181,477 deer annually (mean = 160,050; SD = 16,178). Although the population fluctuated, median population growth rate was 0.99 (90% CRI 0.978–1.003) during the study. Statewide population abundance was estimated at 890,657 (90% CRI 786,627–1,172,514) deer in 2023. Our IPM provided a comprehensive picture of deer population dynamics and allowed us to estimate abundance and demographic rates using only harvest data and informative priors. This model demonstrates the benefits of using informative priors and regularizing parameters in ecological studies. The IPM is a useful, flexible tool to monitor harvested populations at finer spatial scales thereby allowing decisions on harvest regulations to be based on precise estimates of abundance within specific management units.

## Introduction

Population estimates are essential for making decisions for fish and wildlife populations but are often unreliable or too costly to obtain at an appropriate scale, which can hinder effective monitoring and management [1,2]. Management of harvested populations, for instance, relies on accurate assessment of population status within spatially identifiable units (i.e., harvest management units) to reevaluate and establish harvest regulations periodically. Because ecological, biological, and sociocultural factors influencing a species’ population dynamics vary spatially across management units, successful management decisions hinge on reliable estimates of population status at the management unit scale [3–5]. State agencies often collect data from annual harvest, such as total reported harvest or harvest by specific sex and age classes (i.e., age-at-harvest data; [6]). As a result, there are various methods for estimating abundance which are implemented with harvest data including reconstruction models or harvest indices [5,7]. However, traditional methods based solely on harvest data may provide imprecise estimates of abundance at the management unit scale [2,7,8]. For example, the Sex-Age-Kill (SAK) method is sensitive to model violations and fails to provide reliable estimates at the harvest management unit scale [8]. Statistical population reconstruction models often rely on auxiliary data about survival rates, which can be costly to collect [9–11]. Additionally, auxiliary data fragmented across time and space, may constrain the utility of reconstruction models for long-term monitoring.

Integrated population models (IPM) are a flexible tool that use all available data from a population, such as survival data, capture-recapture data, recruitment data, or count data, to provide a comprehensive picture of population dynamics [12,13]. The benefits of an integrated modelling approach include more precise estimates of abundance and demographic rates, explicit consideration of covariation of demographic rates, and parameters without explicit data can often be estimated [12–15]. Integrated population models are hierarchical state-space models that separate the ecological process model and a set of observation models [12,13,16]. The ecological process model is a population model that defines how demographic parameters connect to each other. Observation models connect data sources to demographic parameters and provide a link from the data to the process model [13,14]. This formal separation of the observation and ecological process model allow estimation of both observation and process error [13].

Integrated population models implemented in a Bayesian framework may provide robust estimates of abundance incorporating only harvest data and prior knowledge [11,17]. In a Bayesian framework, there are four basic components: prior distributions that reflect any prior belief in possible parameter values, data, a model that relates the data to the parameters, and posterior distributions that reflect updated belief in parameter values given the prior distributions, data, and model. Most studies use uninformative priors but inclusion of prior information can improve precision and make non-estimable parameters estimable by way of regularization [11,16,18]. Regularizing parameters enhance parameter estimation via smoothing or interpolation and can be achieved by providing informative priors and/or hierarchical parameters with random effects [11]. For example, regularization using hierarchical parameters will predict a grand mean for survival rate among years, and then, with a random year effect and available data, allow survival rates to vary each year. Annual estimates of survival borrow strength across years, and when data are lacking regularization will shrink the estimate toward the grand mean [11,16].

Many harvested populations are well studied, and published information on demographic rates could be used to augment harvest data without integrating additional empirical data to reliably estimate abundance at the management unit scale [11,17]. Norton (2015) developed and tested an age-at-harvest IPM model to estimate abundance of white-tailed deer (*Odocoileus virginianus;* hereafter deer) and found that the model tracked trends in abundance over time well and was not sensitive to errors in recruitment and survival rates [11]. They found that the model was sensitive to initial abundance, but after 3 years the error in abundance was calibrated to (i.e., tracked) the population [11]. Allen et al. (2018) used a similar Bayesian state-space model to estimate a statewide abundance of black bears (*Ursus americanus*) relying solely on harvest data and informative prior distributions based on black bear ecology. Abundance estimates corresponded well with population trends from catch-per-unit-effort and an estimate from capture-recapture data [17]. However, the estimate was statewide and most bears were sexed and aged providing ample harvest data that may not be available for many harvested populations [17]. Estimating abundance within management units instead of statewide reduces sample sizes making it even more difficult to provide reliable estimates [19].

Deer are a widespread and socioeconomically important harvested species throughout North America that are often managed within deer management units (DMUs;[20]). Harvest is the main tool for state agencies to manipulate deer populations and meet management goals, and a critical component of deer management relies on precise estimates of abundance within DMUs. Most states in midwestern and eastern United States use harvest data with accounting or reconstruction models to estimate abundance of deer [2] which may not provide estimates precise enough for management purposes [8,21]. Our objective was to estimate abundance of harvested populations at the scale at which decisions are made for wildlife populations. Specifically, we developed a Bayesian IPM to provide reliable estimates of abundance of harvested populations at the management unit scale relying solely on harvest data. We adapted the model from Norton (2015) to model abundance at the management unit scale and applied the IPM to deer in Tennessee, USA to estimate abundance of deer within DMUs and statewide. Estimates of abundance or density of deer in Tennessee have been limited to either a few counties [22] or to 1 year [23], which limits their utility to inform decisions for harvest management.

## Methods

### Study area

**Fig 1:**
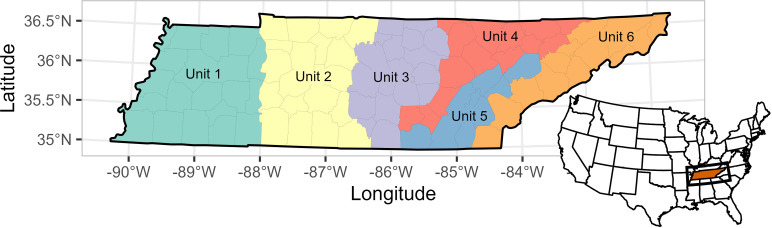
Study area map. Study area for the application of an integrated population model to estimate abundance of white-tailed deer statewide and within the 6 deer management units using harvest data from 2005–2023 in Tennessee, USA.

Tennessee is located in the southeastern United States (34°59′–36°41′ N, 81°39′–90°19′ W) in the Eastern temperate forest ecoregion, and covers roughly 109,150 km^2^ (Fig 1). The state is trisected by the Tennessee River, which forms most of the division between Middle and West Tennessee. Topography transitions from the flat and gently rolling hills of the Mississippi Alluvial Valley in the west to the mountainous terrain of the Appalachian Mountains in the east, and elevation ranges from 54–2,025 m, with a mean of 270 m. The climate was characterized by hot summers and mild to cool winters with an average of 130 cm of precipitation annually. Agricultural production, including cattle grazing, poultry farms, and cropland covered more than 40% of Tennessee, while forests covered roughly 52%. The dominate predator of deer in the region were coyotes (*Canis latrans*), and additional population removal was facilitated though regulated public hunting.

In 2023, Tennessee Wildlife Resources Agency (TWRA) delineated 6 possible DMUs across the state. Hunting regulations changed over time from 2005 to 2023. Aside from antlered deer taken on select public land quota hunts which did not count toward statewide bag limits (i.e., “bonus bucks”), hunters in Tennessee could harvest up to 3 (from 2005–2014) or 2 (from 2015–2023) antlered deer each season under statewide regulations. The antlerless bag limit changed substantially throughout this timeframe, with some counties limited to a weekly county quota for the general gun season, but generally bag limit ranged from 1 in the east up to 3 per day in the middle and west during general gun season. Chronic Wasting Disease (CWD) was first detected in Tennessee in December 2018, prompting the establishment of a new deer hunting unit Unit CWD in western Tennessee; and with it, more liberal hunting regulations such as the Earn-A-Buck and Replacement Buck programs which enabled hunters to take additional antlered deer throughout the season. These programs varied over the years, including an unlimited antlered deer earning system starting in 2020−21 and a reduction in the antlerless deer requirement in 2022−23. Additionally, the antlered bag limit in Unit CWD was increased from two to three antlered deer in the 2022−23 season as the unit expanded to 12 counties. However, as of the 2023−24 hunting season, the newly delineated DMUs were used and Unit CWD was essentially Unit 1.

### Integrated population model structure

**Fig 2:**
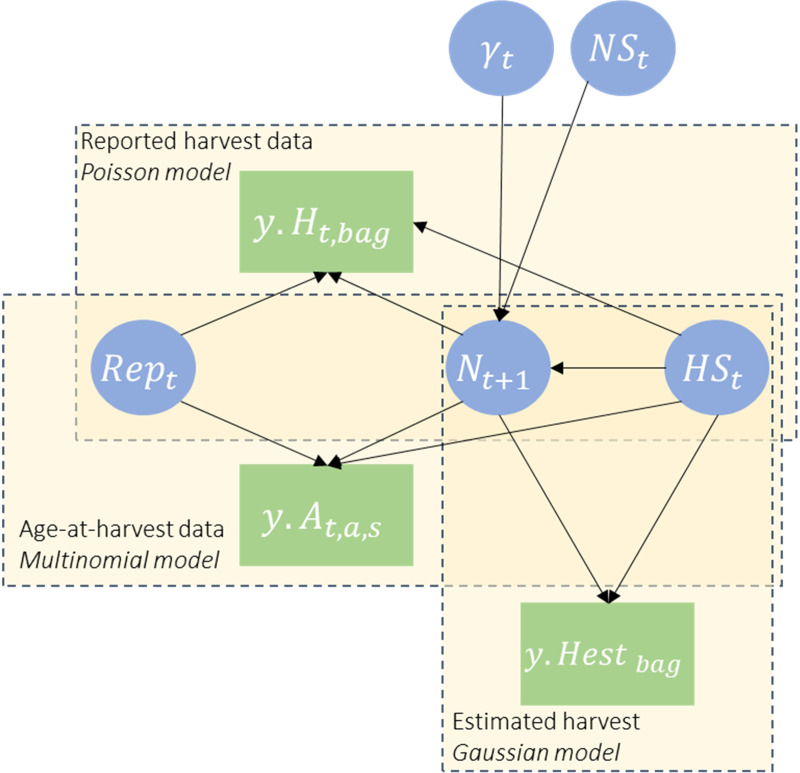
Integrated population model structure. Directed acyclic diagram for an integrated population model for white-tailed deer in Tennessee, USA based on harvest data (green rectangles). The demographic parameters (blue circles) include abundance (N), hunting survival (HS), natural survival (NS), recruitment rate (γ), and reporting rate (Rep).

We developed an IPM to estimate abundance and demographic rates of deer based solely on reported harvest data and total estimated harvest at the management unit and statewide scale (Fig 2). We wanted our IPM to rely on only harvest data, therefore we included additional information in the model using informative priors which is described below.

A state-space model comprised of two process models (the ecological process and observation process) formed the IPM [12–14]. The ecological process model was based on the latent population state process for the pre-hunting season period (mid-fall), which we modeled using a 2 sex, 3 stage (fawn, yearling, adult), post-birth pulse population model [24,25]. In mid-fall, fawns, yearlings, and adults are approximately 0.5, 1.5, and ≥2.5 years old, respectively. Others have similarly used 3 stages for deer population models to account for differences in survival and reproduction (e.g., [26–28]). We assumed population change within DMUs was a function of survival and reproduction, and that immigration and emigration summed to zero and therefore were not included [27]. Although it is possible immigration and emigration could approximately cancel out, it remains important to interpret these demographic rates cautiously as they may be confounded with dispersal [5]. We modeled abundance (N) of yearlings and adults for each sex in each year and DMU as a function of abundance the previous year, hunting survival (HS), and natural survival (NS). As an example, the number of yearlings (a=2) of sex s (s∈{1=females, 2=males}) in year t and DMU i was modeled as


$$N_{2,t,s,i}\;\sim \;Binomial\left(N_{1,t-1,s,i},\;{HS}_{1,t-1,s,i}*{NS}_{1,t-1,s,i}\right)$$ (1)

We used a normal approximation of the binomial distribution which allowed for better convergence and faster run times [29]. Adult abundance was modeled similarly to yearling abundance except that adult abundance depended on both yearling and adult abundance in year t−1. We modeled abundance of fawns as a function of female abundance, recruitment rate (fawns recruited per female; $\gamma_{a,t,i}$), sex ratio (${s.ratio}_{s}$), hunting survival (${HS}_{a,t,s,i}$), and natural survival (${NS}_{a,t,s,i}$) as


$$\begin{array}{c} N_{1,t,s,i}\;\sim \;Poisson\left(\sum_{a=1}^{3}N_{a,t-1,1,i}*{{NS}_{a,t,1,i}}^{\left(\frac{8}{12}\right)}*{HS}_{a,t,1,i}*\gamma_{a,t,i}*{s.ratio}_{s}\right) \end{array}$$ (2)

for age a, sex s, year t, and DMU i. We used a normal approximation of the Poisson distribution for better convergence [29]. We assumed an equal sex ratio at birth (Appendix S1) and the birth pulse was in early June [30,31]. Therefore, females had to survive 8 months, including through the harvest season, to reproduce. Although fawns may enter estrus in the autumn of their first year and successfully reproduce, they rarely breed and to limit parameters we did not include fawn reproduction [32,33]. Pregnancy rates and litter size are connected to maternal age through body condition [32], therefore we allowed recruitment rate to vary by age class (yearlings and adults).

We modeled natural survival, hunting survival, reporting rates, and recruitment using generalized linear models with appropriate link functions. Natural survival (NS), hunting survival (HS), and reporting rates were modeled on the complementary log-log (cloglog) scale, which corresponds to a time-to-event (hazard) formulation and performs better near extremes (i.e., 0 and 1). Specifically, we estimated a baseline, mean hazard for each sex, and included log-hazard ratio (LHR) offsets to account for differences among age classes and a random effect to account for spatiotemporal variability. This structure allowed for flexible age- and sex-specific variation while borrowing strength across ages and among management units and across time [16,27]. Survival rates were modeled using generalized linear mixed-effects models with a logit link function for survival and a log link function for recruitment. For example, natural survival was modeled as:


$$cloglog\left({NS}_{a,t,s,i}\right)={\mu .NS}_{s}+{LHR.NS}_{a}+{\varepsilon .NS}_{t,i}$$ (3)

where μ was the mean parameter value, LHR was the age-specific log-hazard ratio offset, and ε was the random effect for natural survival (NS) for age class a, sex s, year t, and DMU i. Hunting survival (HS) and reporting rates were modeled similarly to equation 3. Because reporting requirements changed drastically over the study, the random effect properly accounted for both spatial and temporal variation. Recruitment rate (γ), defined as the number of fawns produced per female, was modeled on the log scale:


$$\log\left(\gamma_{a,t,i}\right)=\mu .rec+{\varepsilon .rec}_{a,t,i}$$ (4)

where ${\varepsilon .rec}_{a,t,i}$ was a random effect for each year (t), DMU (i), and to account for differences in yearlings (a).

The observation process of our observed harvest data consisted of 2 parts, the total reported harvest (${y.H}_{t,bag,i}$) and the subset of the reported harvest that was aged and sexed (${y.A}_{a,t,s,i}$) for age class a, sex s, and bag∈{Antlerless, Antlered} in year t and DMU i. We computed the total, unobserved harvest ($H_{a,t,s,i}$) and the reported harvest (${Hrep}_{a,t,s,i}$) for age a, sex s, in year t and DMU i as


$$H_{a,t,s,i}=N_{a,t,s,i}*\left(1-{HS}_{a,t,s,i}\right)$$ (5)

$${Hrep}_{a,t,s,i}=H_{a,t,s,i}*R_{a,t,s,i}$$ (6)

where $R_{a,t,s}$ was the reporting rate. The observed total reported harvest data were modeled using a normal approximation of a Poisson whereas the age-at-harvest data were modeled as a multinomial random variable:


$$\begin{array}{c} {y.H}_{t,\;Antlerless,i}\;\sim \;Normal\left(\sum_{a=1}^{3}{Hrep}_{a,t,1,i}+{Hrep}_{1,t,2,i},\sum_{a=1}^{3}{Hrep}_{a,t,1,i}+{Hrep}_{1,t,2,i}\;\right) \end{array}$$ (7)

$$\begin{array}{c} {y.H}_{t,Antlered,i}\;\sim \;Normal\left(\sum_{a=2}^{3}{Hrep}_{a,t,2,i},\sum_{a=2}^{3}{Hrep}_{a,t,2,i}\right) \end{array}$$ (8)

$$P_{a,t,s,i}=\frac{{Hrep}_{a,t,s,i}}{\sum_{a=1}^{3}{Hrep}_{a,t,s,i}}$$ (9)

$$\begin{array}{c} {y.A}_{a,t,s,i}\;\sim \;multinomial\left(P_{a,t,s,i},\;\sum_{a=1}^{3}{y.A}_{a,t,s,i}\right) \end{array}$$ (10)

where $P_{a,t,s,i}$ was the proportion of each age and sex class in the reported harvest. We modeled the observed harvest data statewide by summing the estimated reported harvest by DMU and used equations 7–10 fit to statewide harvest data. We also integrated auxiliary data for estimates of total harvest that were available in 2019 to 2023 as


$$\begin{array}{c} {y.Hest}_{t,Antlerless}\;\sim \;Normal\left(\sum_{a=1}^{3}H_{a,t,Female}+H_{1,t,Male},\sigma_{Hest}\right) \end{array}$$ (11)

$$\begin{array}{c} {y.Hest}_{t,Antlered}\;\sim \;Normal\left(\sum_{a=2}^{3}H_{a,t,Male},\sigma_{Hest}\right) \end{array}$$ (12)

where y.Hest was the estimate of harvest for each year t and bag type, and $\sigma_{Hest}$ was the observation error for total harvest. We calculated population growth rate statewide and by DMU as the ratio of estimated total population size in year t+1 to population size in year t. We calculated mean population growth rate over the study period as the geometric mean of all annual population growth rates.

### Formation of priors

We constructed informative priors for each demographic parameter based on a literature review of deer demographic rates in the southeastern United States (Table 1, S1 Appendix). We used Google Scholar to search for articles that reported vital rates and a measure of variation (e.g., standard errors or confidence intervals) for deer and constrained the results from 2000–2020 for deer primarily in the Southeast or Midwest. For survival rates, we removed seasonal rates and only retained annual estimates for age and sex classes. We then used the Method of Moments [34] to convert reported mean and variances (Table 1, S1 Appendix, S2 Appendix) to the appropriate link scale for our model. Specifically, priors for natural survival, hunting survival, and reporting rates were specified on the complementary log-log scale, while priors for recruitment were specified on the log scale. We assumed random effects followed normal distributions centered at 0 with weakly informative priors on the standard deviations, reflecting observed variation in the literature (Table 1, S2 Appendix). Rather than estimating separate mean parameters for each age and sex class, we modeled survival and reporting rates as hazard functions with a baseline by sex and included age-specific log-hazard ratio offsets that were uninformed, and were normally distributed and centered around 0.

**Table 1:** Prior distributions for an integrated population model. Prior distribution and hyperparameters for a Bayesian integrated population model of white-tailed deer in Tennessee using harvest data. Mean and standard deviation (SD) are specified on the complementary log-log (cloglog) scale for natural survival, hunting survival, and reporting rates, and on the log scale for recruitment rates. Age-specific differences in survival and reporting rates were modeled using log-hazard ratio (LHR) offsets, which were given weakly informative normal priors. Standard deviations for random effects were modeled as normal with mean 0 and SD estimated from Gamma-distributed priors. Prior-posterior overlap (for SD parameter the order is female; male) was used to evaluate priors, and when overlap was > 35% we calculated data-agreement criterion (DAC).

Rate	Parameter	Scale	Mean	SD	Overlap	DAC
Natural survival						
	Female	Cloglog	−2.12	0.235	12.3	NA
	Male	Cloglog	−1.6	0.226	2.4	NA
	SD	Gamma	0.98	14	29.7; 8.7	NA
	LHR Offset (Age)	Cloglog	0	10	NA	NA
Hunting survival						
	Female	Cloglog	−1.8	0.323	37.7	0.41
	Male	Cloglog	−0.96	0.289	37.9	0.55
	SD	Gamma	3.92	28	1.2; 2.5	NA
	LHR Offset (Age)	Cloglog	0	10	NA	NA
Reporting rate						
	Female	Cloglog	−1.5	0.224	33.5	NA
	Male	Cloglog	−1.3	0.218	33.4	NA
	SD	Gamma	2	20	0; 0	NA
	LHR Offset (Age)	Cloglog	0	10	NA	NA
Recruitment rate						
	Mean	Log	−0.35	0.15	1.7	NA
	SD	Gamma	5.12	32	1.1	NA

We evaluated the priors using prior-posterior overlap from the MCMCvis package [35]. We used a threshold of 0.35 to indicate whether there were identifiability issues [36]. However, given we used informative priors and expected there to be overlap, we calculated the data-agreement criterion (DAC) based on the Kullback-Leibler divergence [37]. When DAC was ≤ 1, then the prior is in agreement with the data; however if DAC was > 1 then we widened the prior by inflating the standard deviation by 25% [37].

### Estimating abundance of deer within DMUs

We fit the IPM to harvest data from 2005 to 2023 in Tennessee to estimate abundance of deer statewide and for each DMU. We ran 400,000 iterations with 3 chains, a burn-in of 500,000 iterations, and a thinning rate of 50. We continued to run 400,000 iterations until convergence was reached or a maximum of 5,500,000 iterations. We assessed model convergence using the Gelman-Rubin statistic, $\hat{R}<1.1$ [38], and visual inspection of the MCMC chains and posterior distributions. Posterior distributions were summarized by their median and 90% credible interval (CRI) unless otherwise noted.

We assessed model fit by conducting posterior predictive checks (i.e., Bayesian p-value; [16]. For the estimated harvest and reported harvest by bag type data we used the chi-squared test statistic and for the reported harvest by age and sex class we used the Freeman-Tukey statistic [16,39]. Bayesian p-values close to 0.5 indicate good model fit [16]. Complete code and datasets are available online [40].

## Results

Observed reported statewide harvest ranged from 132,256 to 181,477 (mean = 160,050; SD = 16,178) deer across years. Within DMUs, annual mean reported harvest was 26,674 (SD = 14,951) deer. Deer with known age and sex comprised, on average, 3% (SD = 0.9%) of the harvest each year. Of the subset of harvest that was aged and sexed, 28% (SD = 4.3%) were female and 72% (SD = 4.3%) were male. Fawn females made up 17% of the female harvest that was aged and sexed, whereas yearlings were 26% and adults were 57%. For males, fawns comprised 7%, yearlings were 34%, and adults made up 59% of the male harvest that was aged and sexed.

**Fig 3:**
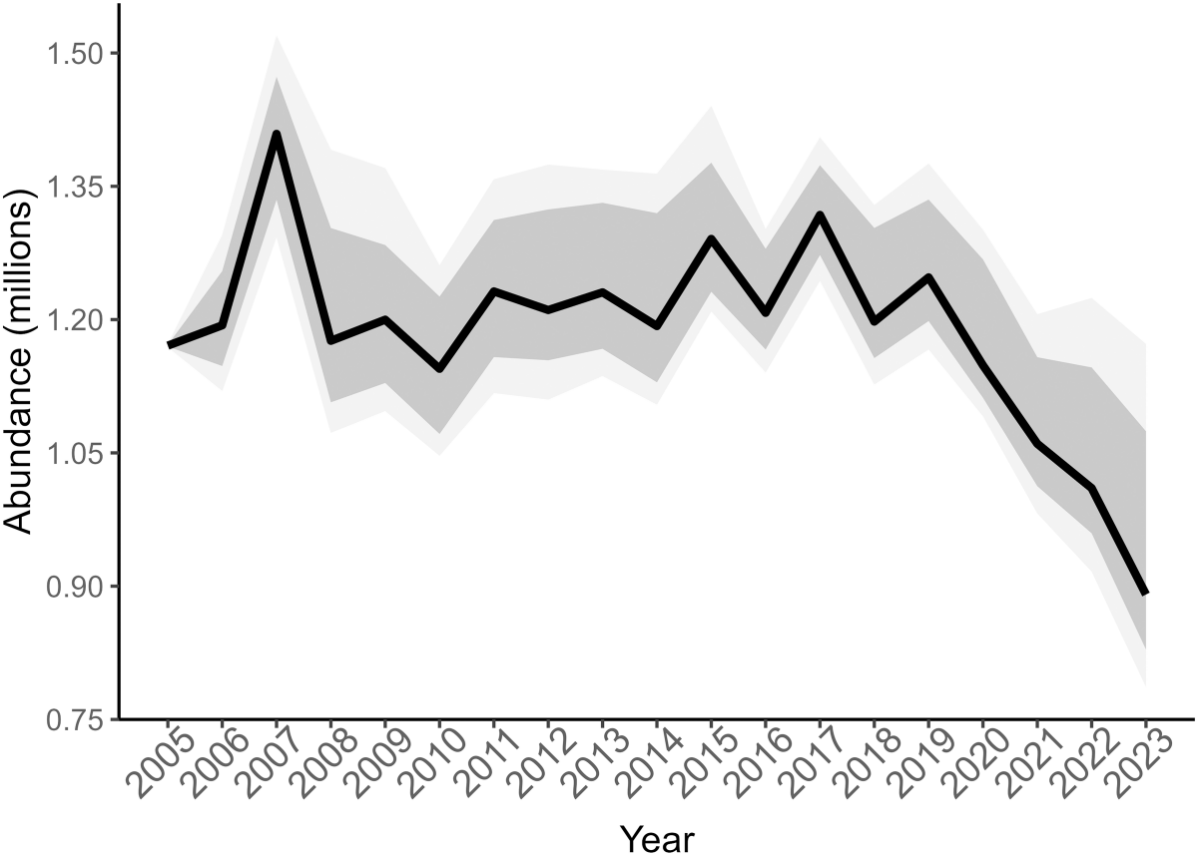
Statewide abundance of deer. Estimated abundance for white-tailed deer in Tennessee from an integrated population model based on harvest data from 2005 to 2023. The black line represents the median estimate, and the shaded areas represent the 66% and 90% Bayesian credible intervals.

**Fig 4:**
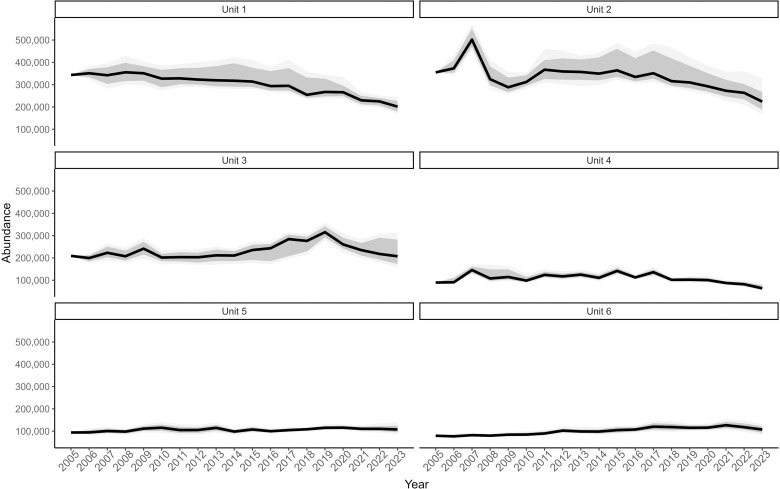
Abundance of deer by management units. Estimated abundance for white-tailed deer in Tennessee, USA by deer management units from an integrated population model based on harvest data from 2005 to 2023. The black line represents the median estimate, and the shaded areas represent the 66% and 90% Bayesian credible intervals.

While the abundance of deer in Tennessee exhibited fluctuations over time, the population remained relatively stable from 2005 to 2023 (Fig 3), with a median population growth rate of 0.99 (90% CRI 0.978–1.003) over 19 years. The population grew by about 1% per year from 2005 to 2019, and then decreased by about 7% per year from 2019 to 2023. The coefficient of variation (CV) for estimates of abundance ranged from 0.33% to 34%, with a mean of 15%. Median proportion of adult and yearling females in the population was 0.35 (90% CRI 0.277–0.408), and 0.29 (90% CRI 0.192–0.314) for adult and yearling males. Statewide abundance was estimated at 890,657 (90% CRI 786,627–1,172,514) deer, which is around 8 (90% CRI 7.2–10.7) deer per km^2^, in 2023. Abundance estimates and uncertainty varied across DMUs, with the greatest abundance and uncertainty in Unit 1 and Unit 2 (Fig 4). In 2023, median abundance in DMUs was 151,891 deer in 2023, and ranged from 63,796 to 223,868 deer (Table 2). Based on the median population growth rate, the population decreased from 2005 to 2023 in Unit 1 (0.97, 90% CRI = 0.961–0.981), Unit 2 (0.98, 90% CRI = 0.959–0.996), and Unit 4 (0.98, 90% CRI = 0.969–0.998) but was stable or increasing in Unit 3 (1.00, 90% CRI = 0.984–1.020), Unit 5 (1.01, 90% CRI = 0.996–1.020), and Unit 6 (1.02, 90% CRI = 1.000–1.030). For abundance estimates within DMUs, CV ranged from 0.17% to 23%, with a mean CV of 11%.

**Table 2:** Abundance of deer by management unit in 2023. Median abundance of deer and density (deer per km^2^) with 90% credible intervals (CRI) for 2023 from an integrated population model based on harvest data from 2005 to 2023 by deer management unit (DMU) in Tennessee.

DMU	Abundance (90% CRI)	Density (90% CRI)
1	201,366 (168,913–243,935)	7 (6.1–8.8)
2	223,868 (167,047–328,320)	9 (6.8–13.4)
3	207,382 (157,636–314,098)	12 (9.3–18.6)
4	63,796 (51,124–87,093)	5 (3.6–6.2)
5	107,635 (87,282–139,741)	12 (9.7–15.8)
6	107,301 (83,379–132,158)	7 (5.6–8.9)

**Fig 5:**
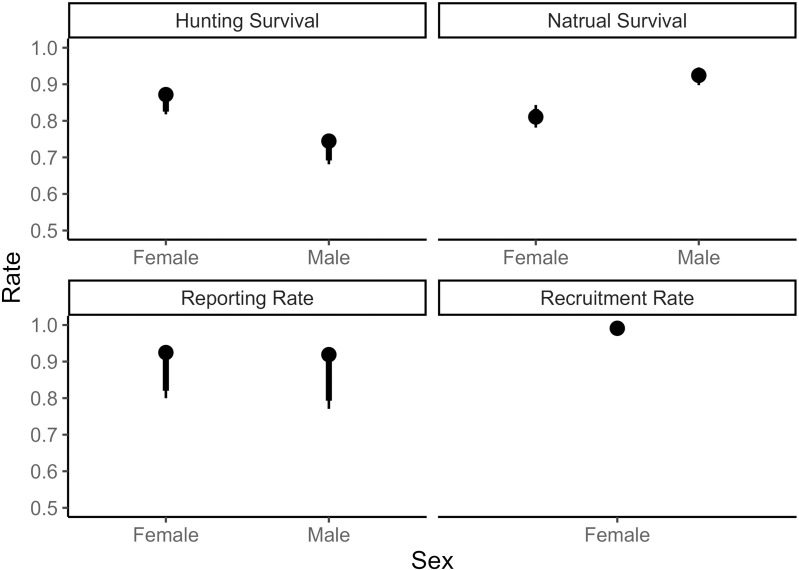
Demographic rates. Estimates of baseline demographic rates from an integrated population model for adult white-tailed deer in Tennessee, USA from 2005 to 2023. Estimates are reported as the median with 66% and 90% Bayesian credible intervals for the baseline or grand-mean parameter value. Demographic rates then varied spatiotemporally from the baseline with random effects and by age with a log-hazard ratio offset. It is important to note that recruitment rate does not yet take into account female survival.

Median reporting rate was similar for adult females than males (Fig 5), and the median baseline reporting rate was 0.93 (90% CRI 0.800–0.941) for females and 0.91 (90% CRI 0.771–0.936) for males. Baseline hunting season survival was lower for males (Fig 5), with a median rate of 0.74 (90% CRI 0.681–0.765; CV = 11%) for adults. Median baseline hunting season survival for adult females was 0.87 (90% CRI 0.818–0.880), and the estimates were precise (CV = 9%). After accounting for spatiotemporal variation and age with the LHR, fawns had the greatest hunting season survival and was similar between males and females at a median value of 0.94 (90% CRI 0.919–0.964). For adult males, median hunting season survival was greatest in DMU 2 (0.77; 90% CRI 0.625–0.87), and was lowest in DMU 6 (0.68; 90% CRI 0.500–0.758). Baseline natural survival was precise (CV < 8%) and slight greater for adult males than adult females (Fig 5). Median natural survival of fawns (0.67; 90% CRI 0.391–0.854) and yearlings (0.98; 90% CRI 0.953–1.00) was similar for males and females across DMUs and time. Baseline survival rates for adult females and males were similar across DMUs, and the median rate was 0.81 (90% CRI 0.607–0.915) and 0.93 (90% CRI 0.619–0.988), respectively. Baseline recruitment rate (fawns recruited per female before accounting for female survival) was 1.2 (90% CRI = 1.04–1.29) whereas fertility (that accounts for survival) ranged from 0.67 (90% CRI 0.607–0.721) in 2018 to 1.24 (90% CRI 1.11–1.43) in 2007.

The IPM fit the harvest data moderately well (S2 Appendix). The fit for antlerless age-at-harvest data had a Bayesian p-value of 0.18, and had the poorest fit among the datasets. The antlered age-at-harvest dataset had a Bayesian p-value of 0.40. The reported harvest by bag type fit the data very well, with a Bayesian p-value of 0.52. The estimated total harvest dataset had a Bayesian p-value of 0.79 indicating moderate fit.

## Discussion

Reliable estimates of abundance and demographic rates are critical for monitoring and management of wildlife populations. Although these population vital rates are critical to making informed decisions and measuring the efficacy of management actions data can be costly to collect and may not provide reliable estimates at the management unit scale. We adapted a Bayesian integrated population model to estimate abundance and demographic rates of harvested populations relying solely on harvest data typically collected by state agencies. We fit this model to harvest data for deer in Tennessee to estimate population dynamics statewide and within deer management units. At a statewide scale, our IPM suggested a stationary population from 2005 to 2023 in Tennessee. However, the population fluctuated year to year with periods of growth and decline. The population was more stable in DMUs with historically more conservative harvest regulations (Units 4, 5, and 6) than DMUs with more liberal regulations (Unit 1 and 2). Our model provides increasing precision and abundance estimates at both statewide and harvest management unit scales than other methods estimating abundance from harvest data [2,7,8]. For example, Millspaugh et al. (2006) evaluated the SAK model for estimating abundance of deer in Wisconsin and found a coefficient of error of ~61% at the DMU level whereas our mean CV was 15% at the DMU level. Granted, the size of the DMUs will affect sample sizes which ultimately affect precision, and the 16 DMUs in Wisconsin were all smaller than the 6 DMUs in Tennessee (see discussion below). Although other studies have found Bayesian state-space models using age-at-harvest data are robust to bias in prior distributions and provide precise estimates [11,17], our study extends the utility of these models to situations with fewer data and provides estimates at multiple scales.

A strength of our IPM was the ability to precisely estimate abundance at multiple spatial scales with only harvest data. Estimates of abundance at the harvest management unit level are needed to evaluate the efficacy of management actions geared at addressing spatially heterogenous issues like productivity or disease and making state-dependent decisions for harvest regulations. Our model provided estimates of abundance that Robson and Reiger (1964) determined were acceptable for rough management (management surveys where a rough idea of population size and composition is needed; [21]), and the mean CV was 15%, which is near the level of precision deemed acceptable for accurate management (<12.5%) and within the level of precision for rough management (<25%; [21]). Further, we provided precise estimates of abundance without expensive auxiliary data, such as radio-tagged data, that is often required with methods relying solely on harvest data [7,9,41,42].

Across DMUs, we observed notable spatial and temporal variation in population trends, underscoring the value of estimating dynamics at management-relevant spatial scales. In particular, Unit 1 showed a sustained decline in abundance estimates beginning in 2010. While interannual fluctuations are common and often influenced by factors such as mast availability, weather conditions, and predation [43,44], the consistent downward trend in Unit 1 aligns with the emergence of chronic wasting disease (CWD) in that region. CWD was first detected in Tennessee in late 2018, and although population-level effects are often delayed, disease-related mortality combined with liberalized harvest regulations in CWD management zones may be contributing to observed declines [45,46]. In response to CWD detection, Tennessee Wildlife Resources Agency increased harvest opportunities in affected areas, including removing antler restrictions and allowing multiple buck harvests [47], which could intensify harvest pressure and influence demographic trends. Additionally, changes in deer movement behavior in response to disease or shifts in hunter participation due to CWD-related concerns may also impact harvest patterns [48,49]. Meanwhile, population trends in other DMUs may reflect variation in resource conditions (e.g., mast production) or stochastic climatic events that influence survival and fawn recruitment [43]. Understanding the full extent of these influences will require further investigation integrating disease surveillance, harvest metrics, and habitat data at finer spatial and temporal resolutions.

A benefit of the IPM over other methods to estimate abundance using harvest data is that IPMs do not assume a stable age distribution. The SAK model, for example, assumes a stable and stationary population [8]. Instead, age structure in the IPM varies through time informed by the data. Further, our IPM offers a comprehensive picture of population dynamics and allowed us to estimate abundance and sex- and age-specific survival, harvest mortality, recruitment, and reporting rates. We found high natural survival rates for fawns (0.5 to 1.5 years old), yearlings, and adults, which is common among ungulate species [50], which were within range of previous estimates (S1 Appendix). Importantly, our findings demonstrate the variability of reporting rate across time and space by both age and sex classes. Many methods to estimate abundance of harvested populations rely on strong assumptions of constant reporting rates [2,7]. With our model structure we were able to account for changes in reporting requirements. Although in general reporting rates for females and were similar across reporting periods, reporting rates for 2020–2023 were greater than the other periods. Additionally, baseline reporting rates for fawn males increased overtime. Using other methods, the violation of the assumption of constant reporting rates would have biased estimates of abundance. Even with standardized reporting requirements, reporting rates change over time and differ by age and sex [51] and our IPM was able to allow reporting rates to vary spatiotemporally within different reporting periods which increased precision and reliability of all estimates from the IPM.

The potential to integrate other data sources or update the model with additional covariates makes this IPM potentially useful for a wide variety of other harvested populations. Additional data for abundance, survival, harvest mortality, recruitment, or reporting rates could all be integrated and improve precision and estimation of abundance and demographic rates [12,13]. Additional data sources need not cover every management unit to be useful because information is shared across space and time. Although the IPM was designed for deer population dynamics, alternative population models could easily be specified for any harvested species. Many management agencies already collect sex and age data for harvested animals, and this model could easily be updated to the specific life-history of the species.

It is important to note that the scale at which units are aggregated for management can influence not only the cost of management but also the information produced from monitoring programs. The scale of harvest management units should be related to an area large enough to determine the effect of management actions while also small and numerous enough to address spatial variation in the habitat quality, human dimensions, and animal population dynamics with desired precision [4,52,53]. Although smaller management units are generally more homogeneous in their habitat and population characteristics, estimates of population dynamics at that scale may be less reliable. In smaller management units it can be difficult to obtain sufficient sample sizes needed to accurately estimate abundance of smaller populations [54]. Further, some methods for estimating abundance using harvest data have proved unreliable at smaller spatial scales due to sampling error and stochasticity [8]. Smaller management units also may have greater movement of individuals between surrounding management units. Not only does this violate assumptions of population closure required by most methods for estimating population dynamics [5], but also increases the influence of management in neighboring units on population objectives in any given management unit.

Oftentimes, managers want to predict future population size under different management scenarios. The model structure easily facilitates predictions of future population size through the use of random effects for spatiotemporal variation. Random effects can be used to extend the scope of inference beyond the samples years or DMUs of our study [16]. Our IPM can therefore be used in a decision-making framework to evaluate the effects of different harvest regulations and provide transparency in management decisions. Manipulation of harvest season survival and the resulting change in abundance or population growth rate can be used to compare different harvest management strategies either informally or formally through structured decision making and adaptive harvest management [55–57]. Beyond evaluating potential management actions, IPMs can be used to test hypotheses about factors affecting population dynamics, such as diseases. Our IPM provides a useful, flexible tool to monitor harvested populations at finer spatial scales thereby allowing decisions on harvest regulations to be based on precise estimates of abundance within specific management units. Importantly, our model can be adjusted to any harvest system and include any additional data sources to further improve estimation of abundance and population dynamics.

## Supporting information

Appendix S1 Literature review. Literature review of white-tailed deer demographic parameters.
(DOCX)

Appendix S2 Model priors. Prior specifications for white-tailed deer integrated population model.
(DOCX)
