# Supplementary material for: Estimating abundance of harvested populations at the management unit scale
Source: PLoS One. 2025 Jun 18;20(6):e0326454. doi: 10.1371/journal.pone.0326454 (PMC12176222; doi:10.1371/journal.pone.0326454)
Supplement: Appendix S1 — Literature review of white-tailed deer demographic parameters. (DOCX) [file pone.0326454.s001.docx]

**Appendix S1 – Literature review of white-tailed deer demographic parameters**

Keever, A. C., Kelly, J. D., Clevinger, G. B., & Cohen, B. S. Estimating abundance of harvested populations at the management unit scale. *PLOS ONE*

*.*

Table S1. Reported estimates for demographic rates of white-tailed deer males and females from a literature review. The literature review was constrained from 2000-2020 for deer primarily in the Southeast or Midwest.

| Rate | Sex | Estimate | State | Source |
| --- | --- | --- | --- | --- |
| Adult birth rate | F | 1.71 (1.67 – 1.75) | MS | [1] |
| Adult birth rate | F | 1.62 | NC | [2] |
| Adult birth rate | F | 1.88 | NC | [2] |
| Adult birth rate | F | 1.82 | AL | [3] |
| Adult birth rate | F | 1.82 (1.80 – 1.84) | MS | [1] |
| Adult birth rate | F | 1.72 (0.89 – 2.55) | IL | [4] |
| Adult recruitment rate | F | 1.12 – 1.92 | AR | [5] |
| Adult hunting mortality | M | 0.508 (0.292 – 0.747) | AL | [6] |
| Adult hunting mortality | F | 0.176 (0.088 – 0.331) | AL | [6] |
| Adult hunting survival | M | 0.54 (0.39 – 0.68) | WI | [7] |
| Adult hunting survival | F | 0.89 (0.85 – 0.93) | WI | [7] |
| Adult hunting survival | M | 0.574 (0.568 – 0.580) | MS | [8] |
| Adult hunting survival | M | 0.55 (0.393 – 0.707) | LA | [9] |
| Adult hunting survival | F | 0.73 | GA | [10] |
| Adult hunting survival | M | 0.85 | GA | [10] |
| Adult hunting survival | M | 0.84 | GA | [10] |
| Adult survival | M | 0.317 (0.150 – 0.515) | AL | [6] |
| Adult survival | F | 0.802 (0.647 – 0.896) | AL | [6] |
| Adult survival | F | 0.87 (0.839 – 0.902) | SC | [11] |
| Adult survival | MF | 0.87 | KY | [12] |
| Adult survival | F | 0.87 | IL | [13] |
| Adult survival | F | 0.83 | IL | [14] |
| Adult survival | F | 0.801 (0.721 – 0.854) | NC | [2] |
| Adult survival | F | 0.88 (0.84 – 0.93) | WV | [15] |
| Adult survival | F | 0.62 (0.43 – 0.79) | IN | [16] |
| Adult survival | M | 0.31 (0.16 – 0.50) | IN | [16] |
| Adult survival | F | 0.71 (0.671 – 0.749) | TX | [17] |
| Adult survival | M | 0.17 | PA | [18] |
| Adult survival | M | 0.59 | PA | [18] |
| Adult survival | M | 0.16 | PA | [18] |
| Adult survival | M | 0.11 | PA | [18] |
| Adult survival | F | 0.59 | PA | [18] |
| Adult survival | F | 1 | PA | [18] |
| Adult survival | F | 0.37 | PA | [18] |
| Adult survival | F | 0.45 | PA | [18] |
| Adult survival | M | 0.527 (0.521 – 0.533) | MS | [8] |
| Adult survival | M | 0.57 | LA | [19] |
| Adult survival | M | 0.77 | GA | [10] |
| Adult survival | F | 0.46 | GA | [10] |
| Adult survival | MF | 0.70 – 0.99 | AR | [5] |
| Adult proportion by sex | M | 0.2 – 0.3 | AR | [5] |
| Adult proportion by sex | F | 0.65 – 0.75 | AR | [5] |
| Adult proportion by sex | M | 0.06 – 0.31 | NY | [20] |
| Adult proportion by sex | F | 0.43 – 0.38 | NY | [20] |
| Fawn birth rate | F | 0.24 (0.075 – 0.405) | IL | [4] |
| Fawn recruitment rate | F | 0 – 0.05 | AR | [5] |
| Fawn/adult survival | F | 0.89 (0.55 – 0.94) | KY | [21] |
| Fawn/adult survival | F | 0.86 (0.78 – 0.96) | KY | [21] |
| Fawn/adult survival | F | 0.91 (0.85 – 0.98) | KY | [21] |
| Fawn/yearling survival | MF | 0.30 – 0.50 | AR | [5] |
| Fawn proportion by sex | M | 0.4 – 0.5 | AR | [5] |
| Fawn proportion by sex | F | 0.125 – 0.175 | AR | [5] |
| Fawn proportion by sex | M | 0.42 – 0.58 | NY | [20] |
| Fawn proportion by sex | F | 0.35 – 0.38 | NY | [20] |
| Neonate survival | MF | 0.43 (0.29 – 0.57) | KY | [22] |
| Neonate survival | MF | 0.318 (0.160 – 0.534) | SC | [23,24] |
| Neonate survival | MF | 0.232 (0.119 – 0.403) | SC | [23,24] |
| Neonate survival | MF | 0.167 (0.071 – 0.343) | SC | [23,24] |
| Neonate survival | MF | 0.513 (0.338 – 0.685) | SC | [23,24] |
| Neonate survival | MF | 0.202 (0.118 – 0.322) | SC | [23,24] |
| Neonate survival | MF | 0.431 (0.294 – 0.581) | SC | [23,24] |
| Neonate survival | MF | 0.185 (0.039 – 0.332) | NC | [25] |
| Neonate survival | MF | 0.105 (0.008 – 0.203) | NC | [25] |
| Neonate survival | F | 0.141 (0.105 – 0.185) | NC | [2] |
| Neonate survival | MF | 0.271 (0.185 – 0.398) | LA | [26] |
| Neonate survival | MF | 0.26 (0.10 – 0.68) | AL | [27] |
| Neonate survival | MF | 0.67 (0.34 – 0.85) | SC | [28] |
| Neonate survival | MF | 0.56 (0.25 – 0.79) | SC | [28] |
| Neonate survival | MF | 0.56 (0.38 – 0.72) | SC | [28] |
| Neonate survival | MF | 0.28 (0.15 – 0.42) | SC | [28] |
| Neonate survival | MF | 0.49 (0.28 – 0.68) | SC | [28] |
| Neonate survival | MF | 0.29 (0.108 – 0.472) | GA | [29] |
| Recruitment | F | 0.58 fawns/doe | KY | [22] |
| Recruitment | MF | 0.288 (0.284 – 0.293) | SC | [23,24] |
| Recruitment | MF | 0.470 (0.468 – 0.472) | SC | [23,24] |
| Recruitment | MF | 0.290 (0.289 – 0.291) | SC | [23,24] |
| Recruitment | MF | 0.652 (0.651 – 0.653) | SC | [23,24] |
| Recruitment | MF | 0.165 (0.164 – 0.166) | SC | [23,24] |
| Recruitment | MF | 0.350 (0.349 – 0.351) | SC | [23,24] |
| Recruitment | MF | 0.174 (0.173 – 0.175) | NC | [25] |
| Recruitment | MF | 0.106 (0.105 – 0.107) | NC | [25] |
| Recruitment | MF | 0.6 | TX | [17] |
| Recruitment | MF | 0.31 | TX | [17] |
| Recruitment | MF | 0.65 | GA | [30] |
| Sex ratio | M | 0.6 | LA | [26] |
| Sex ratio | M | 0.54 | LA | [26] |
| Sex ratio | M | 0.53 | LA | [26] |
| Sex ratio | M | 0.72 | LA | [26] |
| Sex ratio | M | 0.51 (0.49 – 0.52) | IL | [4] |
| Yearling birth rate | F | 1.4 | NC | [2] |
| Yearling birth rate | F | 1.56 | NC | [2] |
| Yearling birth rate | F | 1.13 | AL | [3] |
| Yearling birth rate | F | 1.30 (1.26 – 1.34) | MS | [1] |
| Yearling birth rate | F | 1.4 (0.56 – 2.24) | IL | [4] |
| Yearling recruitment rate | F | 0.58 – 1.11 | AR | [5] |
| Yearling hunting mortality | M | 0.179 (0.070 – 0.402) | AL | [6] |
| Yearling hunting mortality | F | 0.277 (0.077 – 0.567) | AL | [6] |
| Yearling hunting survival | M | 0.81 (0.65 – 0.90) | WI | [7] |
| Yearling hunting survival | F | 0.93 (0.83 – 0.97) | WI | [7] |
| Yearling hunting survival | M | 0.821 (0.817 – 0.825) | MS | [8] |
| Yearling survival | M | 0.821 (0.598 – 0.930) | AL | [6] |
| Yearling survival | F | 0.723 (0.433 – 0.887) | AL | [6] |
| Yearling survival | F | 0.775 (0.630 – 0.880) | NC | [2] |
| Yearling survival | F | 0.86 (0.76 – 0.97) | WV | [15] |
| Yearling survival | M | 0.23 (0.13 – 0.47) | WV | [15] |
| Yearling survival | M | 0.44 | PA | [18] |
| Yearling survival | M | 0.39 | PA | [18] |
| Yearling survival | M | 0.87 | PA | [18] |
| Yearling survival | M | 0.6 | PA | [18] |
| Yearling survival | F | 0.92 | PA | [18] |
| Yearling survival | F | 0.54 | PA | [18] |
| Yearling survival | F | 1 | PA | [18] |
| Yearling survival | F | 1 | PA | [18] |
| Yearling survival | M | 0.821 (0.817 – 0.825) | MS | [8] |
| Yearling proportion by sex | M | 0.3 | AR | [5] |
| Yearling proportion by sex | F | 0.125 – 0.175 | AR | [5] |
| Yearling proportion by sex | M | 0.27 – 0.37 | NY | [20] |
| Yearling proportion by sex | F | 0.22 – 0.24 | NY | [20] |

**REFERENCES**

1. Jones PD, Strickland BK, Demarais S, Blaylock AC. Reproductive characteristics of white-tailed deer in Mississippi. Southeast Nat. 2010;9: 803–812.

2. Chitwood MC, Lashley MA, Kilgo JC, Moorman CE, Deperno CS. White‐tailed deer population dynamics and adult female survival in the presence of a novel predator. J Wildl Manage. 2015;79: 211–219.

3. Turner MA, Gulsby WD, Ditchkoff SS, Gray WN, Cook CW. Effects of breeding chronology on white‐tailed deer productivity in Alabama. Wildl Soc Bull. 2019;43: 701–707.

4. Green ML, Kelly AC, Satterthwaite-Phillips D, Manjerovic MB, Shelton P, Novakofski J, et al. Reproductive characteristics of female white-tailed deer (Odocoileus virginianus) in the Midwestern USA. Theriogenology. 2017;94: 71–78.

5. Collier BA, Krementz DG. Uncertainty in age-specific harvest estimates and consequences for white-tailed deer management. Ecol Modell. 2007;201: 194–204.

6. Wiskirchen K. Survival of adult white-tailed deer and movement relative to temporal patterns of predation risk. Auburn University. 2017.

7. Magle SB, Chamberlin JC, Mathews NE. Survival of white-tailed deer in Wisconsin’s chronic wasting disease zone. Northeast Nat. 2012;19: 67–76.

8. Bowman JL, Jacobson HA, Coggin DS, Heffelfinger JR, Leopold BD. Survival and cause-specific mortality of adult male white-tailed deer managed under the quality deer management paradigm. Proceedings of the Annual Conference of the Southeastern Association of Fish and Wildlife Agencies. 2007. pp. 76–81.

9. Thayer JW, Chamberlain MJ, Durham S. Space use and survival of male white-tailed deer in a bottomland hardwood forest of south-central Louisiana. Proc Annu Conf Southeast Assoc Fish and Wildl Agencies. 2009. pp. 1–6.

10. Stone DB. Foraging behavior, social interactions, and predation risk of white-tailed deer (Odocoileus virginianus) at a concentrated resource. University of Georgia. 2017.

11. Kilgo JC, Vukovich M, Conroy MJ, Ray HS, Ruth C. Factors affecting survival of adult female white‐tailed deer after coyote establishment in South Carolina. Wildl Soc Bull. 2016;40: 747–753.

12. Cox JJ. Community dynamics among reintroduced elk, white-tailed deer, and coyote in southeastern Kentucky. University of Kentucky. 2003.

13. Storm DJ, Nielsen CK, Schauber EM, Woolf A. Space use and survival of white‐tailed deer in an exurban landscape. J Wildl Manage. 2007;71: 1170–1176.

14. Etter DR, Hollis KM, Van Deelen TR, Ludwig DR, Chelsvig JE, Anchor CL, et al. Survival and movements of white-tailed deer in suburban Chicago, Illinois. J Wildl Manage. 2002;66: 500–510.

15. Campbell TA, Laseter BR, Ford WM, Miller K V. Population characteristics of a central Appalachian white‐tailed deer herd. Wildl Soc Bull. 2005;33: 212–221.

16. Clevinger GB. Effect of urbanization on the survival and movements of localized populations of white-tailed deer in southern Indiana. Ball State University. 2017.

17. DeYoung CA, Fulbright TE, Hewitt DG, Wester DB, Draeger DA, DeYoung CA, et al. Synthesis: interactions of white‐tailed deer populations and vegetation in South Texas at different deer densities and nutrition levels. Wildl Monogr. 2019;202: 1–63.

18. Buderman FE. A joint Kaplan-Meier known-fate and Brownie tag-recovery model to estimate harvest and survival rates. Penn State University. 2012.

19. Simoneaux TN, Cohen BS, Cooney EA, Shuman RM, Chamberlain MJ, Miller K V. Fine-scale movements of adult male white-tailed deer in northeastern Louisiana during the hunting season. J Southeast Assoc Fish Wildl Agencies. 2016;3: 210–219.

20. Robinson KF, Fuller AK, Hurst JE, Swift B, Kirsch A, Farquhar J. A structured decision making approach to white-tailed deer buck harvest management in New York State. New York State Dep Environ Conserv Div Fish, Wildl Mar Resour Albany, USA. Albany, New York; 2015.

21. Haymes CA, McDermott JR, Jenkins GSW, Bowling WE, Hast JT, Johannsen KL, et al. Survival and cause‐specific mortality of white‐tailed deer in southeastern Kentucky. J Southeast Assoc Fish Wildl Agencies. 2018;5: 90–96.

22. McDermott JR. Survival and cause-specific mortality of white-tailed deer (Odocoileus virginianus) neonates in a southeastern Kentucky population. University of Kentucky. 2017.

23. Kilgo JC, Ray HS, Vukovich M, Goode MJ, Ruth C. Predation by coyotes on white‐tailed deer neonates in South Carolina. J Wildl Manage. 2012;76: 1420–1430.

24. Kilgo JC, Vukovich M, Scott Ray H, Shaw CE, Ruth C. Coyote removal, understory cover, and survival of white‐tailed deer neonates. J Wildl Manage. 2014;78: 1261–1271.

25. Chitwood MC, Lashley MA, Kilgo JC, Pollock KH, Moorman CE, DePerno CS. Do biological and bedsite characteristics influence survival of neonatal white-tailed deer? PLoS One. 2015;10: e0119070.

26. Shuman RM, Cherry MJ, Simoneaux TN, Dutoit EA, Kilgo JC, Chamberlain MJ, et al. Survival of white‐tailed deer neonates in Louisiana. J Wildl Manage. 2017;81: 834–845.

27. Jackson AM, Ditchkoff SS. Survival estimates of white-tailed deer fawns at Fort Rucker, Alabama. Am Midl Nat. 2013;170: 184–190.

28. McCoy JC, Ditchkoff SS, Raglin JB, Collier BA, Ruth C. Factors influencing survival of white-tailed deer fawns in coastal South Carolina. J Fish Wildl Manag. 2013;4: 280–289.

29. Nelson MA, Cherry MJ, Howze MB, Warren RJ, Conner LM. Coyote and bobcat predation on white-tailed deer fawns in a longleaf pine ecosystem in southwestern Georgia. J Southeast Assoc Fish Wildl Agencies. 2015;2: 208–213.

30. Gulsby WD, Killmaster CH, Bowers JW, Kelly JD, Sacks BN, Statham MJ, et al. White‐tailed deer fawn recruitment before and after experimental coyote removals in central Georgia. Wildl Soc Bull. 2015;39: 248–255.
