# Supplementary material for: Estimating abundance of harvested populations at the management unit scale
Source: PLoS One. 2025 Jun 18;20(6):e0326454. doi: 10.1371/journal.pone.0326454 (PMC12176222; doi:10.1371/journal.pone.0326454)
Supplement: Appendix S2 — Prior specifications for white-tailed deer integrated population model. (DOCX) [file pone.0326454.s002.docx]

**Appendix S2 – Prior specifications and model fit for white-tailed deer integrated population model**

Keever, A. C., Kelly, J. D., Clevinger, G. B., & Cohen, B. S. Estimating abundance of harvested populations at the management unit scale. *PLOS ONE*


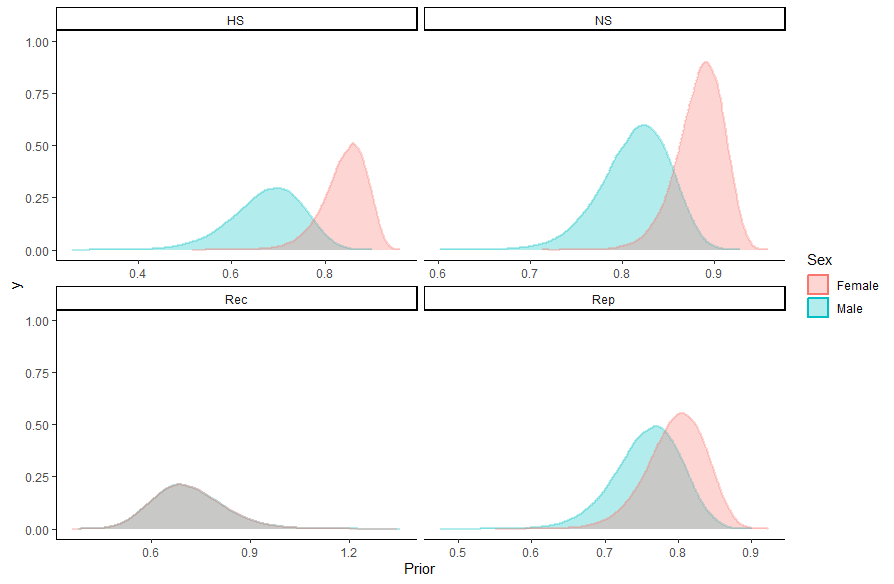


Figure S1. Prior distributions for demographic parameters of adults for a Bayesian integrated population model of white-tailed deer in Tennessee using harvest data from 2005-2023. Parameters include mean hunting survival (HS), mean natural survival (NS), mean recruitment rate (Rec), and mean reporting rate (Rep).


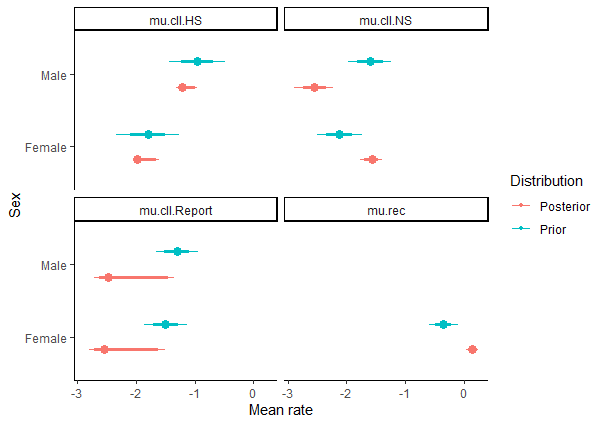


Figure S2. Priors distributions and posterior estimates for hunting season survival (mu.cll.HS), natural survival (mu.cll.NS), reporting rates of harvest (mu.cll.Report) on the cloglog scale, and recruitment (mu.rec) on the log scale for a Bayesian integrated population model of white-tailed deer in Tennessee using harvest data from 2005-2023.


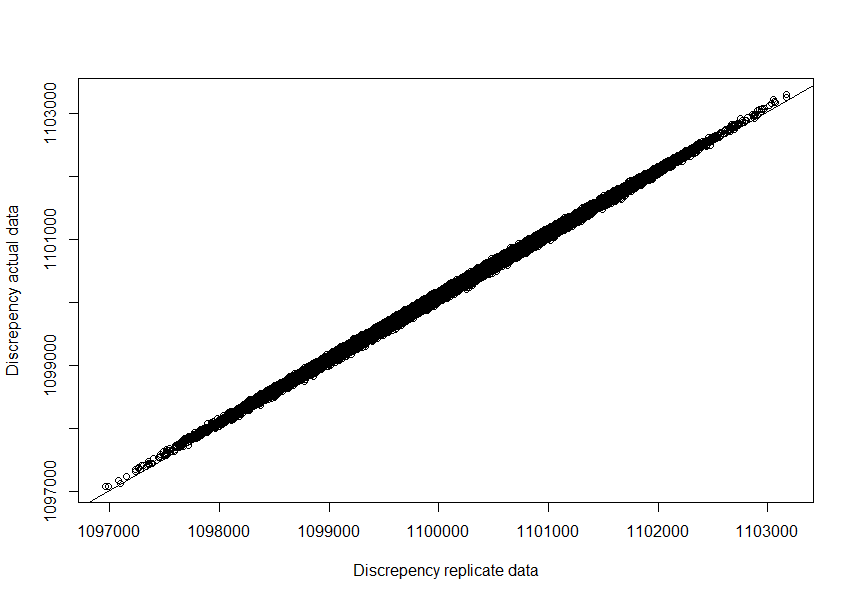


Figure S3. Posterior predictive check of model fit to antlerless age-at-harvest data by scatter plot of the discrepancy measure for replicate (simulated) and actual (observed) data in a Bayesian integrated population model of white-tailed deer in Tennessee using harvest data from 2005-2023


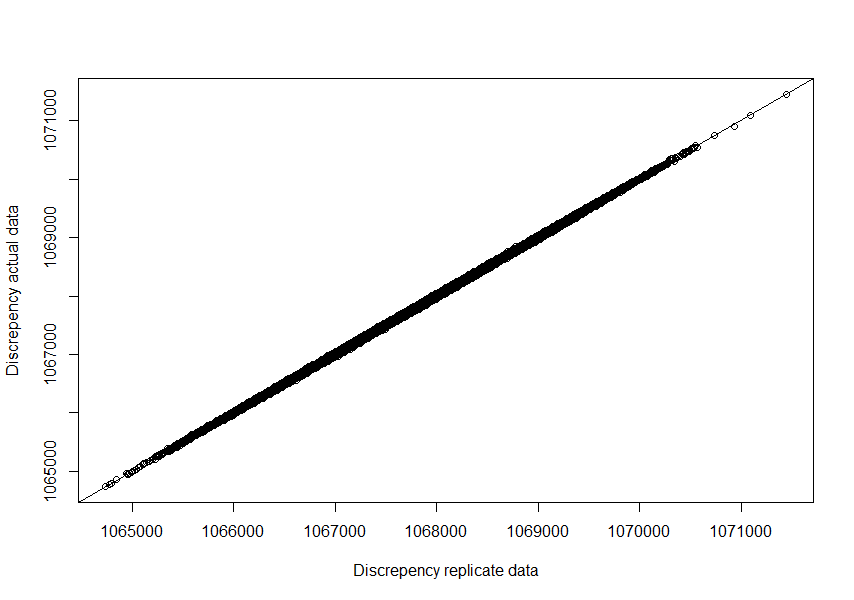


Figure S4. Posterior predictive check of model fit to antlered age-at-harvest data by scatter plot of the discrepancy measure for replicate (simulated) and actual (observed) data in a Bayesian integrated population model of white-tailed deer in Tennessee using harvest data from 2005-2023


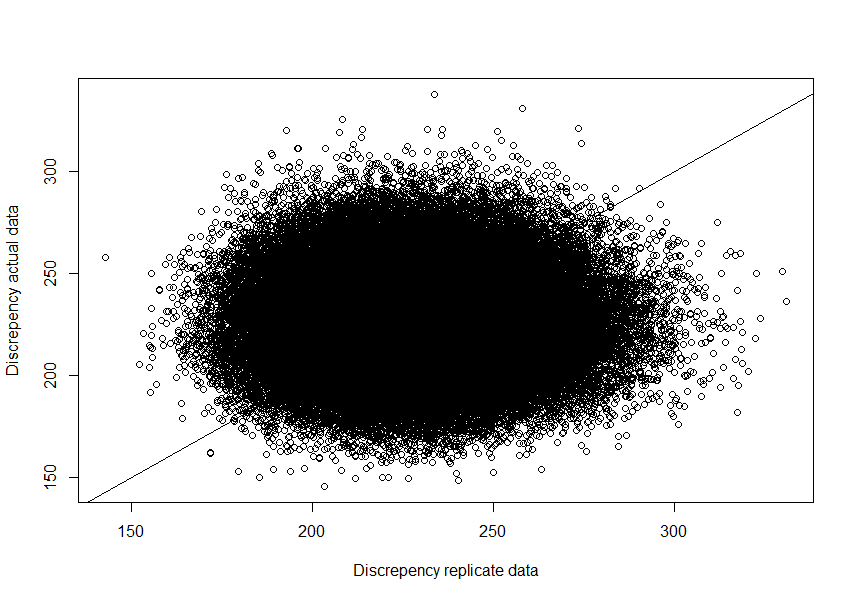


Figure S5. Posterior predictive check of model fit to reported harvest data by scatter plot of the discrepancy measure for replicate (simulated) and actual (observed) data in a Bayesian integrated population model of white-tailed deer in Tennessee using harvest data from 2005-2023


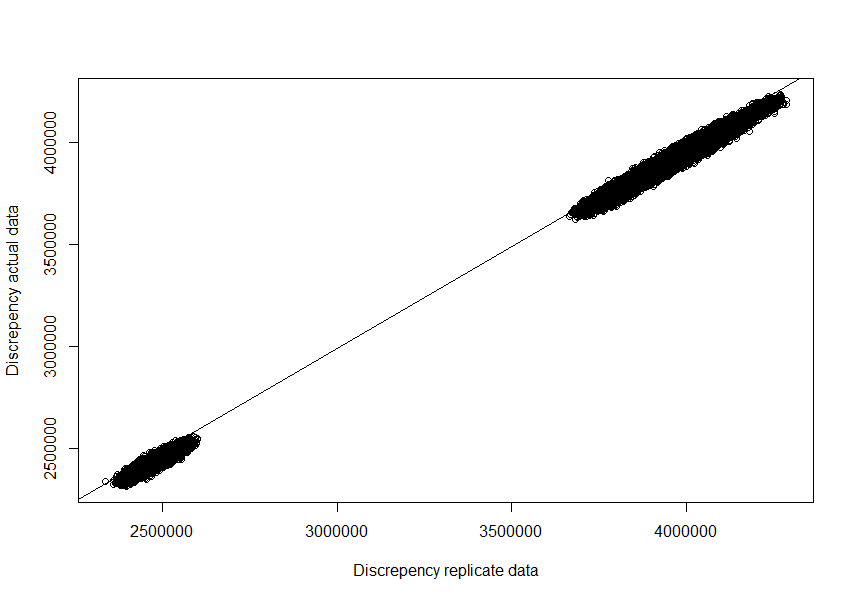


Figure S6. Posterior predictive check of model fit to estimated harvest data by scatter plot of the discrepancy measure for replicate (simulated) and actual (observed) data in a Bayesian integrated population model of white-tailed deer in Tennessee using harvest data from 2005-2023
